# Machine Learning Algorithm Using Electronic Chart-Derived Data to Predict Delirium After Elderly Hip Fracture Surgeries: A Retrospective Case-Control Study

**DOI:** 10.3389/fsurg.2021.634629

**Published:** 2021-07-13

**Authors:** Hong Zhao, Jiaming You, Yuexing Peng, Yi Feng

**Affiliations:** ^1^Department of Anesthesiology, Peking University People's Hospital, Beijing, China; ^2^Key laboratory of Universal Wireless Communication lab, Ministry of Education, Beijing University of Posts and Telecommunications, Beijing, China

**Keywords:** delirium, hip fracture, elderly, machine learning, surgery

## Abstract

**Background:** Elderly patients undergoing hip fracture repair surgery are at increased risk of delirium due to aging, comorbidities, and frailty. But current methods for identifying the high risk of delirium among hospitalized patients have moderate accuracy and require extra questionnaires. Artificial intelligence makes it possible to establish machine learning models that predict incident delirium risk based on electronic health data.

**Methods:** We conducted a retrospective case-control study on elderly patients (≥65 years of age) who received orthopedic repair with hip fracture under spinal or general anesthesia between June 1, 2018, and May 31, 2019. Anesthesia records and medical charts were reviewed to collect demographic, surgical, anesthetic features, and frailty index to explore potential risk factors for postoperative delirium. Delirium was assessed by trained nurses using the Confusion Assessment Method (CAM) every 12 h during the hospital stay. Four machine learning risk models were constructed to predict the incidence of postoperative delirium: random forest, eXtreme Gradient Boosting (XGBoosting), support vector machine (SVM), and multilayer perception (MLP). K-fold cross-validation was deployed to accomplish internal validation and performance evaluation.

**Results:** About 245 patients were included and postoperative delirium affected 12.2% (30/245) of the patients. Multiple logistic regression revealed that dementia/history of stroke [OR 3.063, 95% CI (1.231, 7.624)], blood transfusion [OR 2.631, 95% CI (1.055, 6.559)], and preparation time [OR 1.476, 95% CI (1.170, 1.862)] were associated with postoperative delirium, achieving an area under receiver operating curve (AUC) of 0.779, 95% CI (0.703, 0.856).

The accuracy of machine learning models for predicting the occurrence of postoperative delirium ranged from 83.67 to 87.75%. Machine learning methods detected 16 risk factors contributing to the development of delirium. Preparation time, frailty index uses of vasopressors during the surgery, dementia/history of stroke, duration of surgery, and anesthesia were the six most important risk factors of delirium.

**Conclusion:** Electronic chart-derived machine learning models could generate hospital-specific delirium prediction models and calculate the contribution of risk factors to the occurrence of delirium. Further research is needed to evaluate the significance and applicability of electronic chart-derived machine learning models for the detection risk of delirium in elderly patients undergoing hip fracture repair surgeries.

## Introduction

As life expectancy extends, hip fractures are anticipated to occur from 2.2 to 4.5 million times worldwide annually during the next three decades (1). Surgical repair is always necessary to relieve pain and facilitate early mobilization. Perioperative care needs to focus on providing adequate analgesia and preventing delirium (2, 3). Delirium, one fluctuating condition of consciousness, is common in patients receiving hip fracture repair surgeries with a prevalence of 36.5% (4). It is also associated with prolonged hospitalization, increased 1-year mortality after surgery, poor functional outcomes, high costs, and nursing home placement (5). Prevention strategies are often non-pharmacologic, such as keeping the patients oriented and staying interactive with the environment, which are resource and personnel-intensive (6). If delirium risk could be accurately predicted, prevention could be targeted in high-risk patients to improve patient outcomes and save resources.

Several predictive models were developed to help identify patients with a high risk to develop delirium after hip fracture, but most of them often rely on questionnaires administered by health care professionals (e.g., Mini-Mental State Examination), non-routine clinical data (nursing subjective illness severity assessment), or additional calculations (e.g., Acute Physiology and Chronic Health Evaluation score), which are time and effort consuming and add difficulty in the clinical setting. So far the prediction accuracy of these models is moderate, achieving areas under the receiver operating curve (AUCs) of 0.69–0.81 (7). It is, therefore, important to find more convenient and applicable tools to predict clinical delirium with higher accuracy.

Machine learning is a data analytics technique that uses computational methods to “learn” information directly from data without relying on a predetermined equation as a model. An appropriate machine learning model enables localized specific predictions due to its ability to learn from multiple modules of data and robustness to data noise and could accommodate small size samples of individual hospitals. In addition, machine learning has the potential to analyze underlying mechanisms of different complications (8). Dr. Wong established an electronic health record-based machine learning model to estimate delirium risk in newly hospitalized patients, achieving an accuracy ranging from 0.848 to 0.855 (9).

In this single-center retrospective case-control study, we developed and validated four machine learning models to predict postoperative delirium in elderly patients with hip fractures. These models used clinical variables obtained from hospital charts that automatically predicted the risks for delirium with accuracy ranging from 83.67 to 87.75%.

## Methods

We conducted a retrospective case-control study on elderly patients (≥65 years of age) who received orthopedic repair for low-energy hip fracture under spinal or general anesthesia. Anesthesia records and medical charts were reviewed to collect demographic, surgical, anesthetic features, frailty index, and the occurrence of postoperative delirium. We analyzed the association between these variables and the incidence of delirium to reveal potential risk factors and calculated the prediction accuracies with both multivariate regression and machine learning models. The study was approved by Peking University People's Hospital Institutional Review Board, and informed consent was waived because of the retrospective design. This article complied with the STROBE guidelines for a case-control study.

### Study Protocol

We searched the Anesthesia Information Management System (AIMS) of Peking University People's Hospital for elderly patients (≥65 years of age) undergoing hip fracture repair surgery between June 1, 2018, and May 31, 2019. Diagnosis included fracture of the femoral neck, intertrochanteric, or subtrochanteric fracture. Demographic, surgical, and anesthetic features were extracted from the medical records of patients, and the incidence of postoperative delirium and length of hospital stay were documented.

### Perioperative Management

Early medical optimization and early surgery are the initial goals of care. Simultaneously geriatric pain protocols and a delirium detection program were used throughout hospitalization. All patients were admitted to the orthopedic ward from the emergency department. Tylox® (oral oxycodone 5 mg and acetaminophen 325 mg) was used as the preoperative analgesic; a single-shot of fascia iliaca compartment block (0.3% ropivacaine 30 ml) was performed before surgery in the operating room, then patient-controlled analgesia using sufentanil was given as the postoperative analgesic regimen. Hearing aids and glasses were provided as soon as the patient left the operating room. But interactive activities such as computer games or cognitive rehabilitation were not provided due to shortness of hands.

Upon arrival at the operating room, we established standard monitors for the patients, including ECG, pulse oximetry, and an arterial line. Spinal anesthesia or general anesthesia was left to the discretion of the attending anesthesiologist. Spinal anesthesia was accomplished through L3–L4 intervertebral space with a single dose of 0.5% ropivacaine 2.2–3 ml. General anesthesia was induced with etomidate, cisatracurium, and sufentanil, and a tracheal tube was inserted; the Bispectral index (BIS) was targeted between 40 and 60 during the surgery. All patients who received general anesthesia were extubated in the operating room. Intraoperative hypotension (<90/60 mmHg) was treated with epinephrine or phenylephrine. The criterion to initiate red blood cell transfusion was hemoglobin concentration <9 g•dl^−1^. Those who were with unstable blood pressure or oxygen-dependent were transferred to the intensive care unit (ICU) after surgery for intense monitoring.

### Definition of Variables

Surgical procedures included hemiarthroplasty, total hip arthroplasty, or hollow rivets internal fixation for femoral neck fracture and proximal femoral nail antirotation (PFNA) for intertrochanteric and subtrochanteric fracture. Comorbidity such as dementia, history of stroke, hypertension, diabetes, coronary heart disease, atrial fibrillation, and chronic lung disease was recorded. Demographic, surgical, and anesthetic data were automatically extracted from the AIMS. We used a previously validated chart-derived frailty index, including age >70, preoperative body mass index <18.5, hematocrit <35%, albumin <3.4 g•dl^−1^, and creatinine>176.8 umol•l ^−1^(10). Thereafter, numbers of frailty conditions could be automatically added to achieve a frailty index for each patient.

### Definition of Outcomes

The primary outcome was the incidence of delirium during hospitalization, which was defined as acute, transient, fluctuating, and usually reversible disturbance in attention, cognition, or attention level. Delirium was assessed by trained nurses using the Confusion Assessment Method (CAM) every 12 h during hospital stay until hospital discharge.

### Candidate Predictors

A total of 33 variables were included for candidate predictors, including demographic characteristics, concurrent diseases, surgical data, anesthetic data, consumption of blood products, and preparation time. As dementia and history of stroke are known risk factors for delirium (6), and these two conditions were rare in the study, these two variables were combined to represent compromised brain function at baseline. Preparation time was defined as the calendar days between the diagnosis of hip fracture and surgery. Variables missing in most patients, such as blood gas analysis, were not entered into the logistic regression or machine learning models.

### Cohort Construction and Internal Validation

The dataset was divided into derivation cohort (training set) and validation cohort (test set) with a ratio of 4:1 according to a random number list generated by a computer. K-fold cross-validation method was deployed to accomplish internal validation and performance evaluation. K-fold cross-validation method divides the training dataset into k folds, a classifier is learned using (k-1) folds, and an error value is calculated by testing the classifier in the remaining fold. The k-fold cross-validation estimation of the error is the average value of the errors committed in each fold. The hyperparameter of the proposed model is optimized *via* the k-fold validation method, which can effectively avoid overfitting and underfitting in the case of a small dataset (11).

### Predictive Models

Four risk models were constructed to predict the incidence of postoperative delirium: random forest, eXtreme Gradient Boosting (XGBoost), support vector machine (SVM), and multilayer perception (MLP). Random forest is an ensemble learning method for classification operated by constructing a multitude of decision trees. XGBoost is an implementation of gradient boosted decision trees. SVM are supervised learning models with associated learning algorithms that analyze data used for classification and regression analysis. MLP is an artificial neural network that generates a set of outputs from a set of inputs.

Machine learning methods were also used to analyze the contribution of each variable to the occurrence of delirium, and correlation coefficient, a number between −1 and +1, was calculated to represent the association between the variable and the event. The correlation coefficient was calculated by the layer-wise relevance propagation (LRP) method, which back-propagated relevance recursively from the output layer to the input layer (12, 13). Risk factors with a correlation coefficient >0.5 were considered important predictors for the development of delirium.

All machine learning models were constructed under the Keras framework with Python. Model reporting complied with the Guidelines for Developing and Reporting Machine Learning Predictive Models in Biomedical Research (14).

### Statistical Analysis

Continuous variables are expressed as mean ±SD or median with interquartile range (IQR). Categorical variables are expressed as percentages. All *P*-values were two-tailed, and *P* < 0.05 was considered statistically significant. Statistical analysis was performed using the SPSS 22.0 statistical software package (SPSS Inc., Chicago, IL, U.S.A.). Between-group differences were evaluated using the independent *t*-test or Mann-Whitney U test for continuous variables and the chi-square test or Fisher's exact test for categorical variables, as appropriate. Multivariate logistic regression was adopted to identify risk factors for postoperative delirium.

## Results

**Figure 1:**
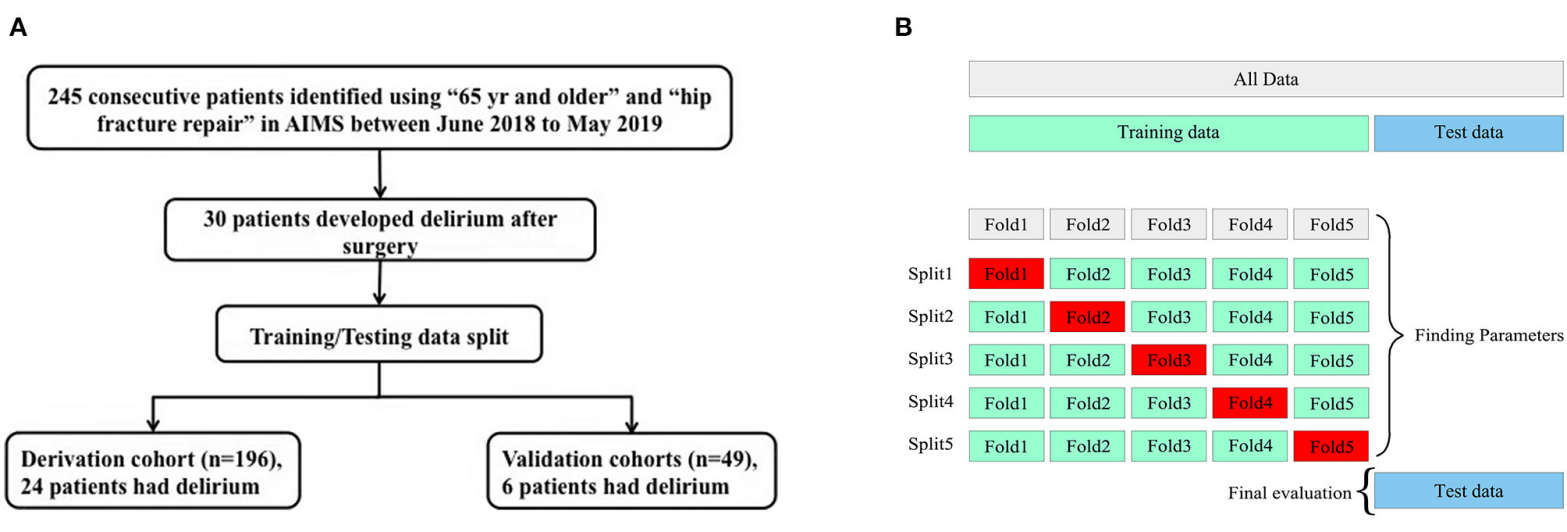
Trial profile and diagram of machine learning model construction. **(A)** Trial profile. **(B)** Diagram of machine learning model construction. AIMS, Anesthesia Information Management System. The complete dataset was split into the training set and test set. The machine learning methods were trained on the training set and applied to the test set for validation and reaching accuracy of prediction. K-fold cross-validation method was deployed (k = 5) to accomplish internal validation and performance evaluation, which divides the training dataset into k-folds, with 39 patients each in fold 1 to fold 4 and 40 patients in fold 5, respectively.

There were 245 consecutive patients identified using “65 years and older” undergoing “hip fracture repair” in AIMS in this study, with the eldest one being 99 years of age (Figure 1A, Table 1). Patients were admitted to the orthopedic ward after diagnosis of hip fracture.

**Table 1:** Univariate and multivariate analysis of perioperative data.

	**Delirium group (*n* = 30)**	**No delirium group (*n* = 215)**	**Statistical value**	***P* value**	**OR**	**95%CI**	
Age (yr)	79.3 (7.6)	80.0 (7.1)	−0.613	0.540			
Male [*n* (%)]	9 (30%)	56 (26.0%)	0.211	0.646			
Height (cm)	159.8 (7.9)	160.0 (8.1)	−0.159	0.874			
Weight (kg)	57.7 (11.1)	57.9 (11.3)	0.032	0.975			
BMI	22.5 (3.8)	22.6 (4.3)	0.10	0.919			
ASA			3.202	0.362			
*1*	0 (0%)	6 (2.8%)					
*2*	21 (70%)	129 (60%)					
*3*	8 (26.7%)	78 (36.3%)					
*4*	1 (3.3%)	2 (0.9%)					
Hypertension	19 (63.3%)	131 (60.9%)	0.064	0.844			
Diabetes	7 (23.3%)	53 (24.7%)	0.025	1.0			
Coronary artery disease	5 (16.7%)	62 (28.8%)	1.963	0.194			
Dementia/History of stroke	12 (40%)	40 (18.6%)	7.208	0.015^*^	3.063	1.231	7.624
Frailty index	2.1 (1.0)	1.9 (1.0)	−0.810	0.418			
Hematocrit	0.32 (0.06)	0.33 (0.06)	−1.235	0.226			
Albumin (g•dl^−1^)	34.8 (4.1)	35.9 (3.6)	1.455	0.147			
Creatinine (umol•l ^−1^)	84 (39)	63 (31)	−0.794	0.427			
Diagnosis			5.478	0.242			
*Fracture of femoral neck*	6 (20%)	75 (34.9%)					
*Intertrochanteric fracture*	19 (63.3%)	116 (53.9%)					
*Subtrochanteric fracture*	5 (16.7%)	24 (11.2%)					
Surgical procedure			2.646	0.266			
*Arthroplasty/Hemiarthroplasty*	4 (13.3%)	48 (22.3%)					
*Hollow rivets*	2 (6.7%)	27 (12.6%)					
*PFNA*	24 (80%)	140 (65.1%)					
General anesthesia [*n* (%)]	8 (26.7%)	25 (11.6%)	5.109	0.04^*^	2.788	0.966	8.047
Blood gas analysis							
*pH*	7.44 (0.04)	7.45 (0.03)	0.961	0.338			
*PaO2*	70 (33.2)	77.4 (14.3)	−0.176	0.861			
Duration of surgery (min)	130 (100)	75 (40)	−2.390	0.017^*^			
Duration of anesthesia (min)	228 (93)	163 (45)	−2.413	0.016^*^	1.009	0.998	1.019
Infused volume (100 ml)	12 (5.63)	9 (5)	−1.924	0.054	0.988	0.914	1.067
Blood loss (ml)	200 (250)	100 (135)	−1.076	0.282			
Intraoperative red cell Infusion (ml)	0 (150)	0 (0)	−2.546	0.011^*^			
Intraoperative fresh frozen plasma infusion (ml)	0 (400)	0 (0)	−3.576	<0.001^*^			
Patients received intraoperative blood products [*n* (%)]	10 (33.3%)	31 (14.4%)	6.759	0.017^*^	2.631	1.055	6.559
Postoperative red cell Infusion (ml)	200 (400)	0 (1000)	−3.813	<0.001^*^			
Postoperative fresh frozen plasma infusion (ml)	0 (400)	0 (0)	−4.012	<0.001^*^			
Patients received postoperative blood products [*n* (%)]	16 (53.3%)	42 (19.5%)	16.6	<0.001^*^			
Preparation time (Days)	3 (2)	2 (3)	−2.822	0.04^*^	1.476	1.170	1.862
Postoperative length of stay (Days)	13 (6)	8 (7)	−2.477	0.013^*^			
Doses of vasopressors	0 (1)	1 (2)	0.354	0.722			
ICU admittance [*n* (%)]	5 (16.6%)	19 (8.8%)	0.177	0.189			
Pneumonia [*n* (%)]	5 (15.1%)	32 (15.0%)	0.0001	0.993			
DVT/PE [*n* (%)]	2 (6.0%)	0 (0%)	14.451	0.017^*^			

*Values are mean (SD) or number (proportion) or median (IQR). PFNA, proximal femoral nail antirotation; Frailty index, the sum of the following frail conditions, age >70, preoperative body mass index <18.5, hematocrit <35%, albumin <3.4 g•dl^−1^, and creatinine>176.8 umol•l ^−1^. Each frailty condition will get one point. DVT, deep venous thrombosis; PE, pulmonary embolism. Preparation time was defined as days between the diagnosis of hip fracture and surgery. Statistical value was t value for t-test of normalized continuous variables, M-W for non-parametric variables, **X**^2^ for the test of categorical variables*.

* *P < 0.05*.

Among the 245 patients, the incidence of postoperative delirium during hospitalization was 12.2% (30/245). All cases of delirium were observed within 48 h after surgery. Patients who developed delirium after surgery had significantly longer postoperative length of hospital stay than those who had no delirium [13 (6) vs. 8 (7) days, *P* = 0.013].

Pneumonia affected 37 (15.1%) patients, two (0.8%) patients developed deep venous thrombosis, and 28 (11.4%) patients were admitted to ICU postoperatively. No patient died during hospitalization (Table 1).

### Multivariate Logistic Regression for the Development of Delirium

**Figure 2:**
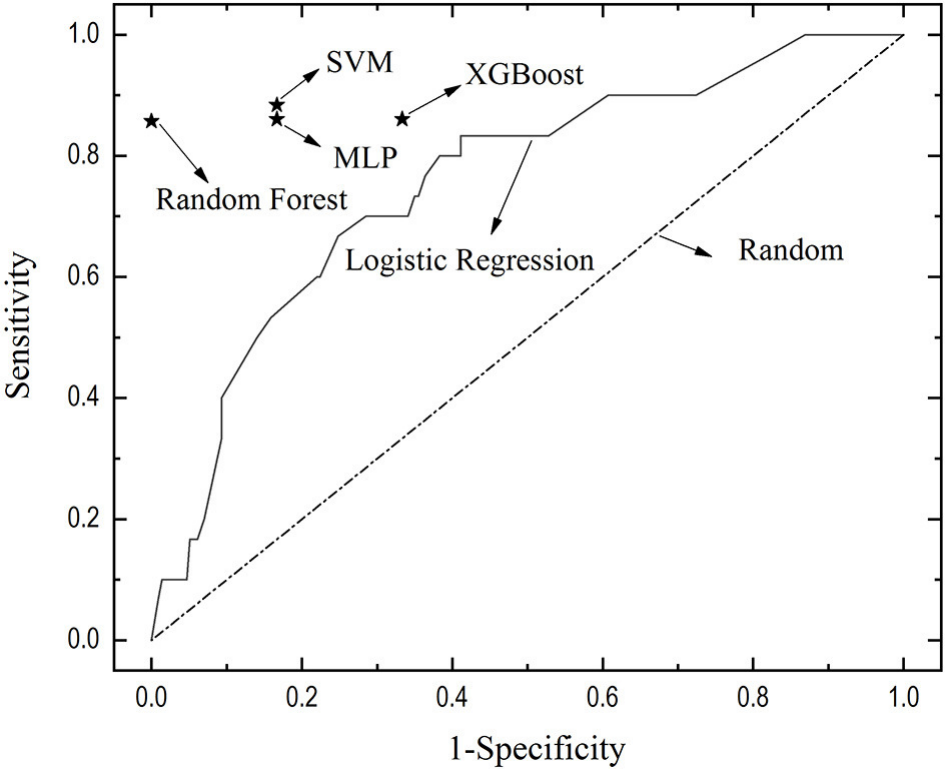
The receiver of the curve of multivariate logistic regression and performance of four machine learning models. The true positive rate-false positive rate of different machine learning models was depicted, locating to the left of and above the ROC curve of logistic regression, indicating the better performance of machine learning models than logistic regression. XGBoosting, eXtreme Gradient Boosting; SVM, support vector machine; MLP, multilayer perception.

Twelve variables were significantly different between the delirium group and non-delirium group, which were dementia/history of stroke, type of anesthesia, duration of anesthesia, duration of surgery, blood transfusion (perioperative volume of red blood cells/fresh frozen plasma transfused and numbers of patients transfused), intraoperative fluid infusion volume, and preparation time prior to the surgery (*P* < 0.1). Six factors entered the multivariate logistic regression, including dementia/history of stroke, type of anesthesia, duration of anesthesia, intraoperative fluid transfusion volume, blood transfusion, and preparation time before surgery. Multiple logistic regression revealed that dementia/history of stroke [OR 3.063, 95% CI (1.231, 7.624)], blood transfusion [OR 2.631, 95% CI (1.055, 6.559)], and preparation time [OR 1.476, 95% CI (1.170, 1.862)] were associated with postoperative delirium (Tables 2, 3), achieving an area under the AUC of 0.779, 95% CI (0.703, 0.856) (Figure 2).

**Table 2:** Multivariate analysis of Postoperative Delirium Preparation time was defined as the calendar days between the diagnosis of hip fracture and surgery.

	**β**	**Standard error**	**Wald**	***P* value**	**OR**	**95%CI**	
Dementia/History of stroke	1.120	0.465	5.792	0.016^*^	3.063	1.231	7.624
General anesthesia	1.025	0.541	3.595	0.058	2.788	0.966	8.047
Duration of Anesthesia (min)	0.009	0.005	2.658	0.103	1.009	0.998	1.019
Intraoperative fluid infusion (ml)	0.000	0.000	0.176	0.675	1.000	0.999	1.001
Patients received blood transfusion	0.967	0.466	4.307	0.038^*^	2.631	1.055	6.559
Preparation time (Days)	0.389	0.119	10.576	0.001^*^	1.476	1.170	1.862

* *P < 0.05*.

**Table 3:** Confusion matrix of machine learning models.

**Models**	**Accuracy**	**False negative**	**True positive**	**False positive**	**True negative**
Random forest	85.71%	16.28%	83.72%	0.00%	100.00%
XGBoost	83.67%	13.95%	86.05%	33.34%	66.66%
SVM	87.75%	11.63%	88.37%	16.67%	83.33%
MLP	85.71%	13.95%	86.05%	16.67%	83.33%

*Accuracy, (True true + true false)/ALL*.

*XGBoosting, eXtreme Gradient Boosting; SVM, support vector machine; MLP, multilayer perception*.

### Machine Learning Model Training and Validation

Four machine learning models were constructed with similar accuracy in predicting postoperative delirium, ranging from 83.67 to 87.75% as shown in the confusion matrix (Table 3). K-fold (k = 5) cross-validation was deployed to accomplish internal validation and performance evaluation (Figure 1B). In this study, the k-fold cross-validation method divided the training dataset into five-folds, a classifier is learned using four-folds, and an error value is calculated by testing the classifier in the remaining fold. The 5-fold cross-validation estimation of the error was the average value of the errors committed in each fold. The hyperparameter of the proposed model was optimized *via* a 5-fold validation method, which can effectively avoid overfitting and underfitting in the case of a small dataset. The true positive rate and false positive rate were also depicted, locating to the left of and above the ROC curve of logistic regression, indicating the better performance of machine learning models than logistic regression (Figure 2).

**Figure 3:**
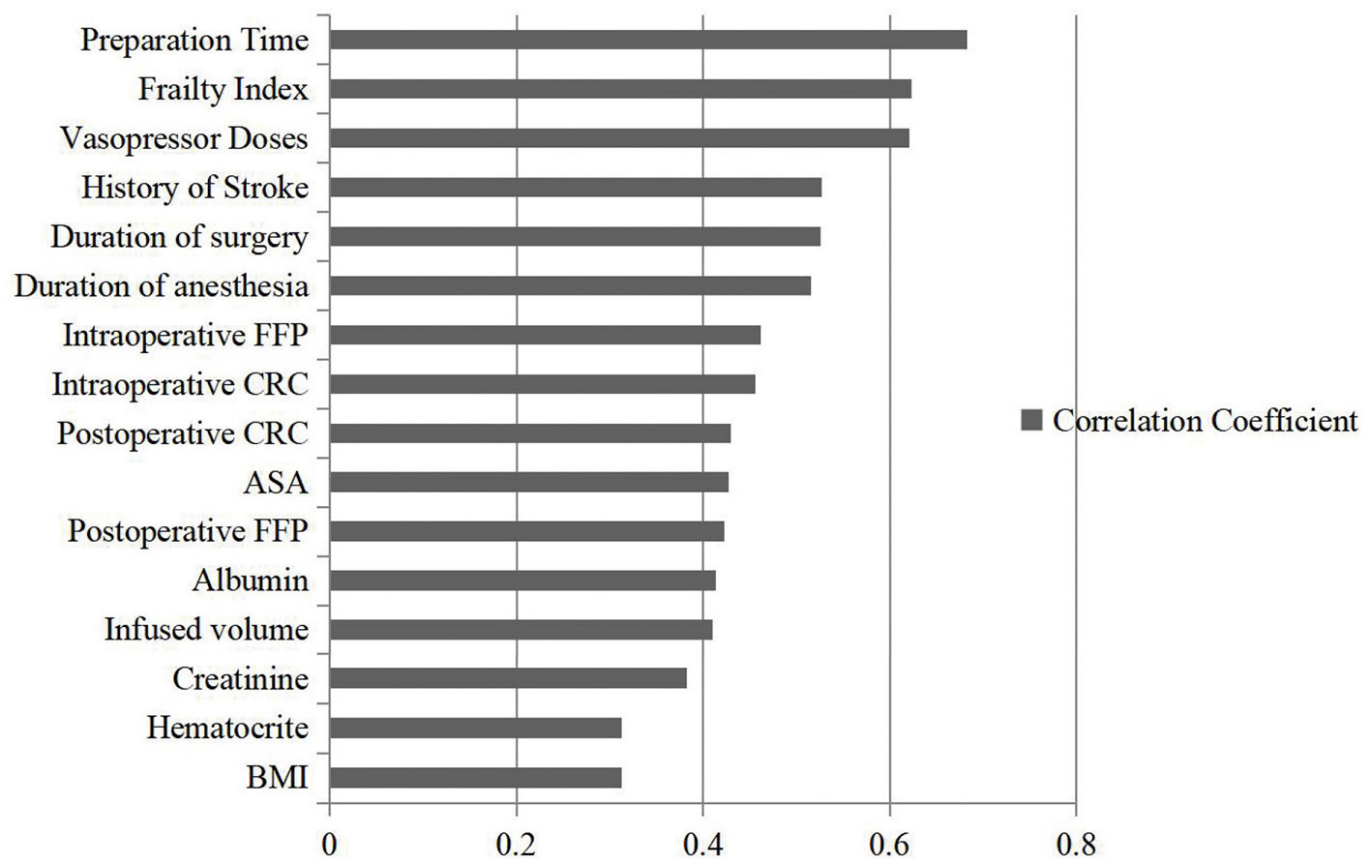
The correlation coefficient of different variables detected by machine learning methods. A correlation coefficient is a number between −1 and +1 calculated to represent the linear dependence of the variable and event. The predictor variable with the largest coefficient is considered as the most important predictor; and the predictor variable with the next largest correlation coefficient as the next important variable, and so on.

The correlation coefficient representing the contribution of each variable to the incidence of postoperative delirium was calculated by the LRP approach. Preparation time, frailty index, use of vasopressors during the surgery, dementia/history of stroke, duration of surgery, and anesthesia were the six most important predictors of delirium (correlation coefficient > 0.5), and their correlation coefficients were present in Figure 3.

## Discussion

In this retrospective case-control study, four machine learning models of delirium prediction were generated using electronic medical charts data, with an accuracy of delirium prediction ranging from 83.67 to 87.75%. Contributions of 16 risk factors to the development of delirium were also detected using machine learning methods. Preparation time, frailty index, use of vasopressors during the surgery, dementia/history of stroke, duration of surgery, and anesthesia were the six most important predictors of delirium after elderly hip fracture surgery.

Hospitals may have individualized and local features that may not allow the implementation of clinical guidelines or significant findings of trials. Machine learning models could provide an opportunity for the integration of individualized and local features in terms of risk estimation, which in turn guides clinical practice. The high accuracies of the machine learning models could be attributed to the fact that they use greater numbers of mathematical operations than traditional regression techniques to better define complex relationships between risk factors and outcomes (9). The four machine learning models are excellent in making accurate predictions out of small-size samples, which makes it more practical in evaluating the hospital-specific risk of hospitalization and mortality (8).

Machine learning models created in this study provide a prediction probability of delirium development with a “snapshot” of chart-derived parameters, which are accurate, objective, and easy to acquire, making them easily incorporated into the hospital workflow (15). Then resource-intense delirium prevention precautions should be applied to high-risk elderly patients with hip fractures (3).

In this study, multivariate logistic regression yielded an AUC of 0.779 in postoperative delirium prediction, which was similar to previous studies (16). Existing predictive models such as the Vochteloo model, used a 9-item model to achieve a score adding formula, with a score of five as a cutoff point (17). AWOL (age >79 years, failure to spell *world* backward, disorientation to place, and higher nurse-rated illness severity) model was another robust tool to predict delirium for newly admitted patients (18). Both models require extra questionnaires and are with moderate accuracy for delirium prediction, ranging from 65 to 75% (16).

Multivariate logistic regression identified dementia/history of stroke, consumption of blood product, and longer preparation time as three risk factors of postoperative delirium for elderly patients with hip fracture. For traditional multivariate logistic regression, at least 5–10 outcome events per input variable have been recommended (19). Dementia/history of stroke could be viewed as a predisposing risk factor for delirium in hospitalized patients (6). However, the other two factors i.e., consumption of blood product and longer preparation time were precipitating factors, which meant they could be adjusted and improved. Studies about blood transfusion conferred controversial results because both anemia and homologous blood transfusion had a detrimental effect on postoperative delirium. Anemia is associated with cerebral hypoperfusion and homologous blood transfusion was a protective factor for delirium patients with the lowest measured hemoglobin level less or equal to 9.7 g•dl^−1^ (20). Whereas, according to Leuzinger et al., elderly patients with hip fractures who received red blood cells transfusion had a longer length of stay and more infections than patients not transfused (21). Hence, avoiding anemia and establishing a hospital-wide transfusion criterion is crucial in this setting. Based on some international consensus, hip fracture repair surgery should be available 7 days a week (22), but in this center, patients who showed up at the emergency department on weekends sometimes did not receive surgery until Monday.

In contrast, the correlation coefficient of 16 variables with the occurrence of delirium was calculated through machine learning approaches, showing the feasibility of machine learning to better define complex relationships between multiple risk factors and outcomes. In addition to the three risk factors identified by multiple logistic regression, frailty index and intraoperative vasopressor doses were also associated with the occurrence of delirium. Frailty emerged as an important indicator of morbidity and mortality for elderly patients undergoing surgical procedures (23, 24). Intraoperative vasopressor doses might be viewed as a reflection of episodes of hypotension, which were related to the emergence of delirium (25).

Machine learning models have another strength besides high accuracy, using electronic chart-derived parameters and confirmation of correlation coefficient of different risk factors, i.e., the accuracy of machine learning models could be improved over time, as exemplified by improvement in the comparability of diabetic retinopathy grading between ophthalmologists and retina specialists (26, 27). With the widespread use of artificial intelligence and more understanding of delirium, more complicated and accurate prediction is under construction, and more characteristics could be added to the models, such as biomarkers for frailty or neuroinflammation to investigate the underlying mechanism of delirium.

This study had some limitations. First, all variables were obtained from electronic medical records and AIMS, some data may be missing, especially microbiology, radiology, or biomarkers related to the underlying mechanism of delirium. More studies are encouraged to investigate the underlying mechanism for delirium occurrence. Second, some variables such as walking ability before hospital admission or financial status were not documented in the chart. But the chart-derived frailty index could be used as an indicator for the functional capacity of patients.

In conclusion, electronic chart-derived machine learning models (random forest, SVM, XGBoost, and MLP) could generate hospital-specific delirium prediction models and calculate the contribution of risk factors to the occurrence of delirium. Further research is needed to evaluate the significance and applicability of electronic chart-derived machine learning models for the detection risk of delirium in elderly patients undergoing hip fracture repair surgeries.

## Data Availability Statement

The raw data supporting the conclusions of this article will be made available by the authors, without undue reservation.

## Ethics Statement

The studies involving human participants were reviewed and approved by Peking University People's Hospital Institutional Review Board. Written informed consent for participation was not required for this study in accordance with the national legislation and the institutional requirements.

## Author Contributions

HZ helped with study design, the conduct of the study, and manuscript preparation. JY and YP helped with machine model construction. YF helped with the study design. All authors contributed to the article and approved the submitted version.

## Conflict of Interest

The authors declare that the research was conducted in the absence of any commercial or financial relationships that could be construed as a potential conflict of interest.
