# Supplementary material for: Machine Learning Algorithm Using Electronic Chart-Derived Data to Predict Delirium After Elderly Hip Fracture Surgeries: A Retrospective Case-Control Study
Source: Front Surg. 2021 Jul 13;8:634629. doi: 10.3389/fsurg.2021.634629 (PMC8313764; doi:10.3389/fsurg.2021.634629)
Supplement: Supplementary file 1 [file Table_1.DOCX]

Supplement Table 1. Multivariate analysis of Postoperative Delirium

|  | β | Standard error | Wald | P | OR | 95%CI |  |
| --- | --- | --- | --- | --- | --- | --- | --- |
| Dementia/History of stroke | 1.120 | 0.465 | 5.792 | 0.016* | 3.063 | 1.231 | 7.624 |
| General anesthesia | 1.025 | 0.541 | 3.595 | 0.058 | 2.788 | 0.966 | 8.047 |
| Duration of Anesthesia (min) | 0.009 | 0.005 | 2.658 | 0.103 | 1.009 | 0.998 | 1.019 |
| Intraoperative fluid infusion (ml) | 0.000 | 0.000 | 0.176 | 0.675 | 1.000 | 0.999 | 1.001 |
| Patients received blood transfusion | 0.967 | 0.466 | 4.307 | 0.038* | 2.631 | 1.055 | 6.559 |
| Preparation time (Days) | 0.389 | 0.119 | 10.576 | 0.001* | 1.476 | 1.170 | 1.862 |

Preparation time was defined as the calendar days between the diagnosis of hip fracture and surgery.

*P < 0.05
